# *Poria cocos* Ethanol Extract Restores MK-801-Induced Cytoskeleton Regulation in Neuro2A and IMR-32 Cells and Locomotor Hyperactivity in C57BL/6 Mice by Modulating the Rho Signaling Pathway

**DOI:** 10.3390/cimb47050312

**Published:** 2025-04-28

**Authors:** Ya-Ying Chang, Cheng-Wei Lu, Tzu-Yu Lin, I-Shiang Tzeng, Yi-Chyan Chen, Mao-Liang Chen

**Affiliations:** 1 Department of Anesthesiology, Far-Eastern Memorial Hospital, New Taipei City 220216, Taiwan; gingerdoll@gmail.com (Y.-Y.C.); drluchengwei@gmail.com (C.-W.L.); drlin1971@gmail.com (T.-Y.L.); 2 International Program in Engineering for Bachelor, Yuan Ze University, Taoyuan City 320315, Taiwan; 3 Department of Medical Research, Taipei Tzu Chi Hospital, Buddhist Tzu Chi Medical Foundation, New Taipei City 231016, Taiwan; istzeng@gmail.com; 4 Department of Psychiatry, Taipei Tzu Chi Hospital, Buddhist Tzu Chi Medical Foundation, New Taipei City 231016, Taiwan; yichyanc@gmail.com

**Keywords:** *Poria cocos* ethanol extract, MK-801, Rho signaling, F-actin, cell migration, hyperactivity

## Abstract

*Poria cocos* extract attenuates MK-801-induced hyperactivity via RhoA/ROCK1 pathway modulation in mice. Background/Objectives: *Poria cocos* (*P. cocos*), a traditional East Asian medicinal mushroom, serves as a medicine and nutritional supplement, has been used to improve sleep and mood. Its bioactive compounds may regulate calcium signaling and Rho family proteins, which are linked to cytoskeletal remodeling and psychiatric symptoms. This study investigated the effects of *P. cocos* ethanol extract (PCEE) on Rho signaling, cytoskeleton dynamics, and behavior in MK-801-treated cells and mice. Methods: PCEE components were analyzed using HPLC. IMR-32 and Neuro2A cells were treated with MK-801 and PCEE to assess changes in F-actin (via fluorescence staining), cell migration (wound healing and Transwell assays), and Rho signaling proteins (by immunoblotting). In vivo, C57BL/6 mice received MK-801 to induce hyperactivity, followed by PCEE treatment. RhoA/ROCK1 pathway protein levels in the prefrontal cortex were analyzed. Results: PCEE reversed MK-801-induced inhibition of cell migration, F-actin disruption, and dysregulation of Rho-related proteins (RhoGDI1, RhoA, CDC42, Rac1, ROCK1, MLC2, PFN1). In mice, PCEE significantly reduced MK-801-induced hyperactivity and normalized RhoA/ROCK1 signaling in the brain. Conclusion: PCEE modulates cytoskeletal dynamics by regulating RhoA/ROCK1 signaling and attenuates MK-801-induced behavioral and molecular changes, suggesting its therapeutic potential for psychosis with fewer adverse effects.

## 1. Introduction

Psychotic disorders are mental illnesses characterized by various manifestations, including perceptual disturbances, cognitive impairment, and hallucinations [1]. Dysfunction of glutamatergic transmission, especially that of the N-methyl-D-aspartate (NMDA) receptor, contributes to the development of neuropsychiatric pathologies [1,2]. Dysregulated NMDA pathways may lead to neuropsychiatric diseases, such as schizophrenia [2]. MK-801 (or dizocilpine), a noncompetitive NMDA receptor antagonist, has been widely used in animal models of pharmacology-induced psychosis to induce symptoms of schizophrenia [3,4,5]. Locomotor hyperactivity has also been observed in rats with repeated MK-801 exposure related to impaired neuron function [4].

A possible psychosis-associated pathway is Rho guanosine triphosphate hydrolase (GTPase) signaling, which is activated by guanine nucleotide exchange factors and inactivated by GTPase-activating proteins [6]. The activation of Rho family proteins regulates neuronal cell function by regulating cell motility and cytoskeletal arrangements [7,8,9]. Altered cytoskeletal dynamics are also linked to psychotic manifestations. For example, changes in the morphology of neuronal dendritic spines are associated with altered efficacy of synapses and, thus, with learning ability and cognitive function [10]. The most studied proteins in the Rho family are Ras homolog family member A (RhoA), Ras-related C3 botulinum toxin subtract 1 (Rac1), and cell division cycle 42 (CDC42) [11]. All of these proteins are associated with actin regulation and motility [11]. For example, the regulation of actin polymerization and the formation of actin myosin filaments are closely related to RhoA, Rho-associated protein kinase 1 (ROCK1), and myosin light chain 2 (MLC2) [12,13]. In addition, profilin-1 (PFN1), an actin-binding protein, is closely associated with cell migration and axon elongation by modulating Rho family proteins [14,15]. PFN1 induces actin polymerization via the RhoA/ROCK pathway [16]. The Rho GDP-dissociation inhibitor (RhoGDI), which is upstream of RhoA/ROCK1 signaling, controls GTPase activity [17]. Previous reports have shown that increased cytoplasmic calcium leads to the phosphorylation of Rho guanine nucleotide dissociation inhibitor 1 (RhoGDI1) [18], which mediates the activation of RhoA [19].

*Poria cocos* is used in East Asian traditional medicine and also in a nutritional supplement that contains various major ingredients, such as triterpenoids and polysaccharides [20,21]. *P. cocos* is the major component of several widely used herbs, such as *Root Poria* and Indian Bread [20,21]. In clinical practice, *P. cocos* is effective at stabilizing emotions and improving sleep quality [22]. Various reports revealed that calcium level is also modulated by the *P. cocos* and MK-801 in cells [23,24,25]. However, whether *P. cocos* mitigates MK-801-induced psychotic behavioral changes by regulating cytoskeletal dynamics remains unclear. Our previous report revealed that the water extract of *P. cocos* modulates the effects of ketamine (an NMDA receptor antagonist) on glial cell migration and Rho-associated protein expression [26]. In this study, we aimed to investigate the regulatory effects of *P. cocos* on Rho signaling in MK-801-treated IMR-32 and Neuro2A cells and on locomotor hyperactivity and Rho signaling in a mouse model of MK-801-induced psychosis. We also explored the upstream expression and regulation of RhoGDI1. We investigated the role of the RhoA/ROCK1 pathway in psychosis and the potential therapeutic effect of *P. cocos* on psychotic behavioral changes.

## 2. Materials and Methods

### 2.1. Preparation of the P. cocos Ethanol Extract and MK-801

The *P. cocos* concentrated herbal extract (manufactured by Sun Ten Pharmaceutical Co., Ltd., New Taipei City, Taiwan) is an herbal powder that has undergone standard ingredient analysis according to the Taiwan Herbal Pharmacopoeia (4th Edition, published by the Ministry of Health and Welfare of Taiwan) to ensure its medicinal effects. The *P. cocos* concentrated herbal extract (purchased from Sun Ten Pharmaceutical Co., Ltd.) has also been approved as a clinical herbal/complementary medicine by the Taiwan Food and Drug Administration. To prepare the *P. cocos* ethanol extract (PCEE), 250 mg of *P. cocos* concentrated herbal extract was dissolved in 25 mL of 99.5% absolute ethanol at room temperature with gentle shaking for 2 h. The *P. cocos* ethanol solution was centrifuged, and the supernatant was transferred to a fresh 50 mL centrifuge tube. The solution was adjusted to a total volume of 50 mL by adding 99.5% absolute ethanol, transferred to 1.5 mL centrifuge tubes (1 mL/tube), and then freeze-dried for 6 h to remove the ethanol. The dried pellet was redissolved in 1 mL of double-distilled water (ddH_2_O) to form 10 mg/mL PCEE. The PCEE was then stored at −20 °C until further use. MK-801(Biosynth Ltd., Compton, Berkshire, UK) was first dissolved in DMSO and then diluted with ddH_2_O to a 1 mM stock solution for in vitro cell culture study and to a 70 μg/mL stock solution for in vivo animal treatment.

### 2.2. Determination and Quantification of the Components in PCEE by High-Performance Liquid Chromatography (HPLC)

Four standard chemicals, including pachymic acid (Cayman Chemical Company, Ann Arbor, MI, USA), tumulosic acid (Cayman Chemical Company), dehydrotumulosic acid (Cayman Chemical Company), and polyporenic acid C (TargetMol Chemicals Inc. Wellesley Hills, MA, USA), were used to identify the components extracted from *P. cocos*. Each standard was dissolved in DMSO at a final concentration of 10 mg/mL stock solution and stored at −80 °C until used. The stock solution of each standard was diluted with methanol to a 50 μg/mL standard solution in HPLC detection. Four standards were mixed to form a mixed standard (100 μg/mL for each of the standards) for evaluating the efficient HPLC separation condition of the four standards. The PCEE was diluted with methanol for analysis. The SHIMADZU (Shimadzu Corporation, Nakagyo-ku, Kyoto, Japan) HPLC system consisted of a DGU-405 online degasser, an LC-40d XR quaternary pump, an SIL-40C XR autoinjector, a CT040S column thermostat, and an SPD-M40 DAD detector. The data were acquired and analyzed by the LabSolutions software. The chromatographic experiments were conducted on a shim-pack GIST C18 column (250 mm, 4.6 mm, 5 mm, obtained from SHIMADZU) under gradient elution at 30 °C. The mobile phase used for the HPLC analysis was composed of acetonitrile (ACN) (A) and water containing 0.05% H_3_PO_4_ (*v*/*v*) (B). The time program was as follows: 0–3 min, A-B (40:60, *v*/*v*); 3–25 min, gradually changing to A-B (85:15, *v*/*v*); 25–30 min, A-B (85:15, *v*/*v*). The column was then flushed with ACN for 1 min and equilibrated with the initial composition for 4 min prior to the next injection. The flow rate was constant at 1.0 mL/min and the injection volume was 10 µL. The UV detection wavelengths were set to 210 nm for PA and TA, and 243 nm for DTA and PPA. ACN (BAKER ANALYZED HPLC ULTRA Gradient Solvent, J.T. Baker™, Thermo Fisher Scientific Inc., Waltham, MA, USA) was purchased from Thermo Fisher Scientific Inc., and the H_3_PO_4_ (85%) was of analytical grade and was purchased from Merk (Sigma-Aldrich Chemie GmbH, Taufkirchen, Germany). The concentrations of 100, 50, 10, 5, and 1 g/mL of each standard chemical were used to generate a standard curve for quantifying TA, PA, DTA, and PPA in PCEE. A measure of 10 μL 100 mg/mL PCEE in methanol was injected into the HPLC for quantification. The quantity of TA, PA, DTA, or PPA in PCEE was determined using the standard curve accordingly.

### 2.3. Cell Culture and Cell Stimulation

To clarify whether there is a difference in drug effects induced by MK-801 and PCEE in the regulation of cell function in neuronal cells of different species, human neuroblastoma IMR-32 cells and mouse neuroblastoma Neuro2A cells were used to perform the experiments in this study. Human neuroblastoma IMR-32 cells and mouse neuroblastoma Neuro2A cells were obtained from the Bioresource Collection and Research Center of the Food Industry Research and Development Institute, Taiwan. IMR-32 and Neuro2A cells were cultured in minimal essential medium supplemented with 10% fetal bovine serum (Gibco, Life Technology Corporation, Frederick, MD, USA), 0.1 mM non-essential amino acids (Gibco, Life Technology Corporation), 2 mM L-glutamine (Gibco, Life Technology Corporation), and 1 mM sodium pyruvate (Gibco, Life Technology Corporation) in a humidified atmosphere of 5% CO_2_ at 37 °C. The cells were passaged, as required for maintenance, upon reaching approximately 80% confluence. Cells of fewer than 12 passages were used in this experiment.

**Figure 1:**
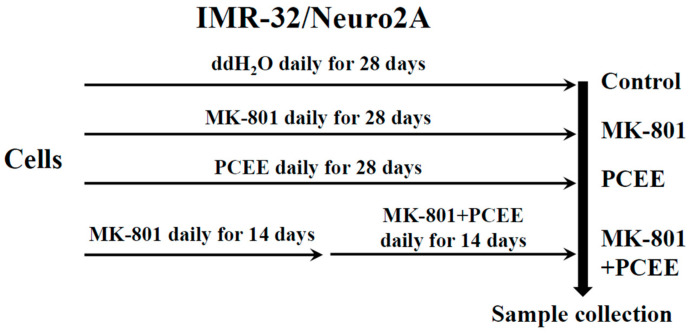
In vitro experimental protocol. IMR-32 and Neuro2A cells were seeded into 10 cm culture dishes and divided into control, MK-801, PCEE, and MK-801 + PCEE groups. The cells in the MK-801, PCEE, or MK-801 + PCEE groups were treated with MK-801, PCEE, or MK-801 in combination with PCEE, respectively, for 28 days. ddH_2_O was added to the cells in the control group.

IMR-32 and Neuro2A cells were seeded into 10 cm culture dishes and divided into control, MK-801, PCEE, and MK-801 + PCEE groups (Figure 1). In the groups treated with MK-801, the cells were stimulated with MK-801 (Sigma–Aldrich, St. Louis, MO, USA) daily for 28 days. For the groups treated with PCEE, PCEE was added to the cells daily for 28 days. To examine the effect of PCEE on MK-801-treated cells, the cells were treated with MK-801 alone daily for 14 days and then with a combination of MK-801 and PCEE daily for another 14 days. The in vitro experimental protocol is shown in Figure 1, and the cells were treated with a final concentration of 1 μM MK-801 or/and 10 μg/mL PCEE.

### 2.4. Animal Preparation and Experimental Protocol

**Figure 2:**
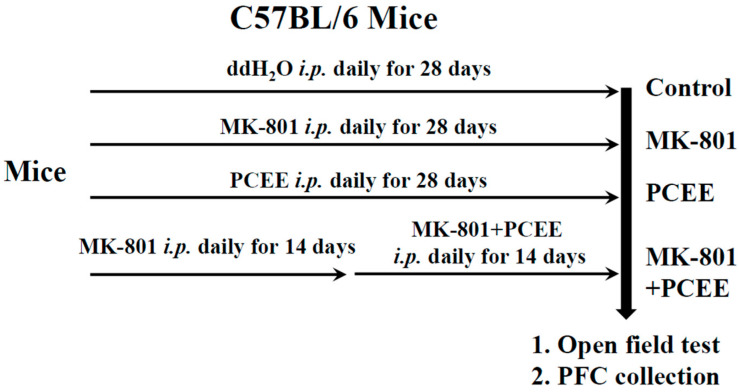
In vivo experimental protocol. C57BL/6 mice were randomly allocated to the control, MK-801, Poria cocos ethanol extract (PCEE), or MK-801 + PCEE groups (n = 3 per group). The mice in the control, MK-801, or PCEE groups were *i.p.* injected with ddH_2_O, MK-801, or PCEE, respectively, for 28 days. The mice in the MK-801 + PCEE group were *i.p.* injected with MK-801 for 14 days and then with MK-801 plus PCEE for another 14 days.

All animal experiments and experimental procedures were approved by the Institutional Animal Care and Use Committee (IACUC) of Taipei Tzu Chi Hospital, Buddhist Tzu Chi Medical Foundation (IACUC-111-028). Six-week-old male C57BL/6 mice were housed in an individually ventilated cage (IVC) system with a 12 h light/12 h dark cycle. The average temperature in the room was maintained 24 ± 1 °C, and the average humidity was maintained at 50%, with free access to food and water. In the groups stimulated with MK-801, the mice were intraperitoneally (*i.p.*) injected with MK-801 daily for 28 days. In the groups treated with PCEE, the mice were *i.p.* injected with PCEE daily for 28 days. In the groups treated with MK-801 plus PCEE, the mice were *i.p.* injected with MK-801 daily for 14 days and then with MK-801 plus PCEE for another 14 days. The mice in the control group were *i.p.* injected with ddH_2_O for 28 days. The mice were randomly divided into control, MK-801, PCEE, and MK-801 + PCEE groups (*n* = 3 per group), as shown in Figure 2. After the behavioral tests (Open Field Test) were conducted, the mice were sacrificed. The prefrontal cortex (PFC) was quickly dissected, frozen in liquid nitrogen for 2 h, and then stored at −80 °C until use. The in vivo experimental protocol is illustrated in Figure 2, and the mice were treated with a final amount of 0.35 mg/kg/day MK-801 [27,28] or/and 10 mg/kg/day PCEE.

### 2.5. Wound Healing Assay and Transwell Migration Assay

A wound healing assay was performed to measure cell mobility. The cells from each group were seeded in 24-well plates with culture inserts (Ibidi GmbH, Gräfelfing, Germany) and cultured to form cell-free gaps. After the cell-free gap formed, the cells were left to grow with MK-801 or/and PCEE for 24 h. The wounded regions of interest were analyzed via light microscopy. The Transwell migration assays were conducted using a 24-well Transwell system (Corning^®^, New York, NY, USA) with microporous polycarbonate membranes (8 µm pores size). The cells were seeded into the medium with MK-801 or/and PCEE in the upper inserts at a density of 5 × 10^3^ cells/mL and allowed to migrate for 24 h at 37 °C. The membranes with migrated cells at the bottom were subsequently sliced from Transwell, washed with 1× phosphate-buffered saline (PBS), fixed with methanol, and stained with propidium iodide solution (50 μg/mL) (Sigma, St. Louis, MO, USA) for 30 min. The migrated cells were counted using an immunofluorescence microscope. Three independent assay batches were performed for statistical analysis for each experimental group.

### 2.6. Total Protein Extraction and Immunoblotting

The expression of Rho pathway-associated proteins was determined by immunoblotting. In brief, cell lysates were prepared using CelLytic™ M Cell Lysis Reagent (Sigma-Aldrich, St. Louis, MO, USA) with protease and phosphatase inhibitors (Thermo Scientific). The protein levels of the cell lysates in each group were quantified via the Bio-Rad Protein Assay Dye Reagent (Bio-Rad Laboratories, Inc., Hercules, CA, USA). Protein extracts (10–50 μg) were loaded on 8% or 12.5% sodium dodecyl sulfate–polyacrylamide gels, separated, and then transferred to polyvinylidene fluoride (PVDF) membranes. The membranes were blocked with 5% skim milk for 1 h, incubated with specific primary antibodies, and then incubated with a secondary antibody (1:10,000; horseradish peroxidase-conjugated anti-mouse IgG antibody; Amersham Pharmacia Biotech, Inc., GE Healthcare., Buckinghamshire, UK). A Western Lightning^®^ Plus-ECL kit (PerkinElmer, Inc., Waltham, MA, USA) was used to visualize the protein bands on the membranes. ImageJ 1.54 g software was used to quantify the density of the protein bands. The expression of each protein is presented as a ratio to that of β-actin and was calibrated relative to that of the control group. The primary antibodies used in this study were as follows: anti-β actin (GTX26276, GeneTex Inc., Alton Pkwy Irvine, CA, USA); anti-RhoGDI1 (#2564, Cell Signaling Technology, Inc., Danvers, MA, USA); anti-RhoGDI (phospho S174) (ab74142, Abcam Limited, Cambridge, UK); anti-RhoA (67B9) (#2117, Cell Signaling Technology); anti-p-RhoA (Ser188) (sc-32954, Santa Cruz Biotechnology, Inc., Santa Cruz, CA, USA); anti-CDC42 (#2462, Cell Signaling Technology), anti-p-CDC42 (ab74142, Abcam Limited, Cambridge, UK); anti-Rac1 (GTX100761, GeneTex Inc.); anti-Rac1 (phospho S71, Abnova Corporation, Taipei, Taiwan); anti-ROCK1 (ab45171, Abcam); anti-Myosin Light Chain 2 (#3672, Cell Signaling Technology); anti-p-MLC2 (anti-MYL2 phospho Ser-18) (TA309976, OriGene Technologies, Inc., Rockville, MD, USA); and anti-profillin-1 (#3237, Cell Signaling Technology).

### 2.7. Fluorescence Staining of Activated RhoGDI1 and F-Actin

Neuro2A and IMR-32 cells were treated with PCEE or MK-801 for 7 days and then treated with either PCEE or MK-801 or both for an additional 5 days. The drug-treated Neuro2A and IMR-32 cells were then transferred to 6-well plates with poly-L-lysine coated coverslips and cultured with PCEE, MK-801, or their combination for another 2 days. The drug-treated cells were fixed and permeabilized with 1% paraformaldehyde and methanol. To detect the expression of p-RhoGDI1 on the cell membrane, an anti-p-RhoGDI1 antibody and an Alexa Fluor^®^ 488 donkey anti-rabbit polyclonal antibody (Abcam, Waltham, MA, USA) were used to probe the activated Rho GDI1. To examine F-actin reorganization, cells on coverslips were stained for 60 min using the CytoPainter Phalloidin-iFluor 488 reagent (Abcam, Waltham, MA, USA). The coverslips were sealed using Slow-Fade™ Diamond Antifade Mountant (Waltham, MA, USA) (with DAPI) on glass slides, and visualized under a fluorescence microscope at 40× magnification.

### 2.8. Open Field Test

C57BL/6 mice (*n* = 3 per group) were subjected to a 30 min locomotor activity assay via an open field test 30 min after the last drug treatment. The mice were allowed to familiarize themselves with the environment for the first 10 min in an open field. The locomotor activity of the mice was examined, and the distance, time, and speed of movement were recorded. The behavior of the mice was evaluated in the first 10–20 min and for a total of 0–30 min.

### 2.9. Statistical Analysis

One-way ANOVA analysis was applied for statistical analysis to compare the differences between groups (control, MK-801, PCEE, MK-801+ PCEE) in Western blot, cell migration assay, and wound healing assay in this study. *p* values of <0.05 (*) and <0.01 (**) were defined as statistically significant.

## 3. Results

### 3.1. HPLC Confirmed PCEE Contained the Main Components of P. cocos

**Figure 3:**
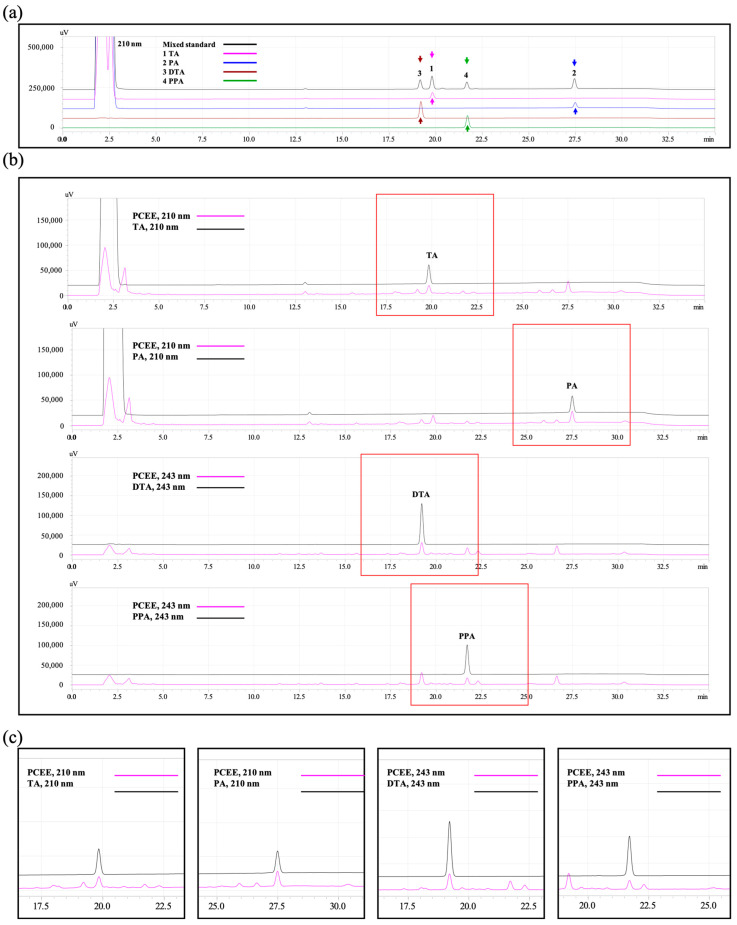
The HPLC chromatograms of the ingredients of *P. cocos*. A mixture of the four standards and individual chromatograms of each standard (**a**) was displayed. The retention times of 1. TA (pink arrow), 2. PA (blue arrow), 3. DTA (brown arrow), and 4. PPA (green arrow) is indicated as shown. HPLC profiles of reference standards were presented in (**b**,**c**). PA, pachymic acid; TA, tumulosic acid; DTA, dehydrotumulosic acid; PPA, polyporenic acid C.

By using HPLC, we verified the efficient separation of the four standards according to the chromatograms of the retention time of the four-standard mix. As shown in Figure 3a, the four standards can be detected and separated in the HPLC analysis according to the retention time (RT) of DTA (RT = 19.2 min); TA (RT = 19.8 min); PPA (RT = 21.7 min); and PA (RT = 27.5 min). We further revealed the HPLC of TA and PA. We also verified the presences of the main ingredients (PA, TA, DTA, and PPA) in PCEE (Figure 3b,c). The results showed that the method used to prepare PCEE can effectively extract the main ingredients from *P. cocos.*

The key compounds (TA, PA, DTA, and PPA) were further quantified using HPLC. The quantity of key compounds was shown in Table 1. The corresponding quantities of TA, PA, DTA, and PPA are 16.803, 29.343, 11.772, and 8.692 μg in PCEE made from 100 mg *P. cocos* concentrated powder.

### 3.2. PCEE Modulates the Expression and Phosphorylation of RhoA, CDC42, and Rac1

**Figure 4:**
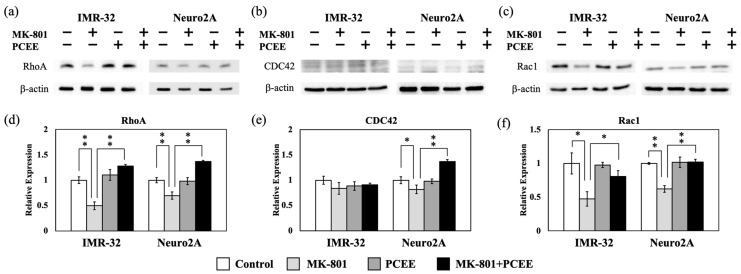
Effects of MK-801 and PCEE on the expression of Rho family proteins in IMR-32 and Neuro2A cells. Protein extracts collected from IMR-32 and Neuro2A cells treated with MK-801 and/or PCEE were examined for the expression of (**a**) RhoA, (**b**) CDC42, and (**c**) Rac1, and the quantitative results are shown in (**d**–**f**). The bar charts were generated from triplicate Western blot data from three different batches of drug-treated cells. A *p* value less than 0.01 (**) or 0.05 (*) from ANOVA followed by Dunnett’s test was considered significant.

Rho family proteins, including RhoA, CDC42, and Rac1, are important regulators of Rho signaling that modulate various cellular functions, such as cytoskeleton remodeling, cell mobility, and cell shape. Therefore, an in vitro study was proceeded to investigate the effects of MK-801 and/or PCEE on Rho family proteins expression and phosphorylation. MK-801 downregulated RhoA expression (Figure 4a,d) in IMR-32 and Neuro2A cells. This inhibitory effect of MK-801 on RhoA expression was significantly reversed by PCEE (*p* value < 0.01). In Neuro2A cells, the inhibitory effect of MK-801 on CDC42 expression was reversed by PCEE treatment (Figure 4b,e). We did not observe any significant effect of PCEE on CDC42 expression in IMR-32 cells (Figure 4b,e). Rac1 is another widely studied Rho family protein related to skeletal dynamics [29,30]. MK-801 downregulated Rac1 expression in IMR-32 and Neuro2A cells, and this effect was reversed by PCEE (Figure 4c,f).

**Figure 5:**
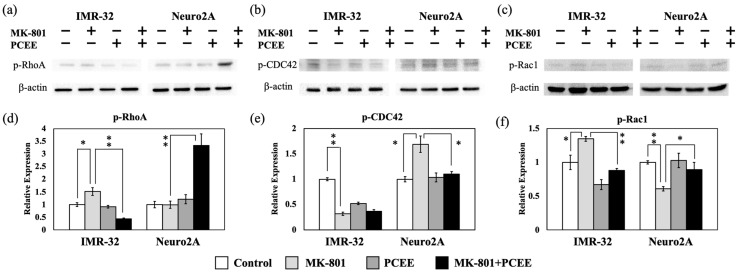
Effects of MK-801 and PCEE on the phosphorylation of Rho family proteins in IMR-32 and Neuro2A cells. Protein extracts collected from IMR-32 and Neuro2A cells treated with MK-801 and/or PCEE were examined for the expression of (**a**) phosphorylated RhoA (p-RhoA), (**b**) phosphorylated CDC42 (p-CDC42), and (**c**) phosphorylated Rac1 (p-Rac1) in IMR-32 and Neuro2A cells. The quantitative results are shown in (**d**–**f**), respectively. The bar charts were generated from triplicate Western blot data from three different batches of drug-treated cells. A *p* value less than 0.01 (**) or 0.05 (*) from ANOVA followed by Dunnett’s test was considered significant.

Furthermore, PCEE significantly counteracted the effects of MK-801 on RhoA phosphorylation in IMR-32 cells (*p* value < 0.01) (Figure 5a,d). Treatment with MK-801 plus PCEE increased RhoA phosphorylation in Neuro2A cells (Figure 5a,d). MK-801 significantly increased the level of p-CDC42 (*p* value < 0.05), and this effect was counteracted by PCEE in Neuro2A cells (Figure 5b,e). In IMR-32 cells, PCEE did not significantly reverse the ability of MK-801 to reduce CDC42 phosphorylation (Figure 5b,e). Consistent with the Rac1 expression data, PCEE significantly reversed the effect of MK-801 on Rac1 phosphorylation in IMR-32 (*p* value < 0.01) and Neuro2A (*p* value < 0.05) cells (Figure 5c,f).

### 3.3. PCEE Inhibits the MK-801-Induced Phosphorylation of RhoGDI1

**Figure 6:**
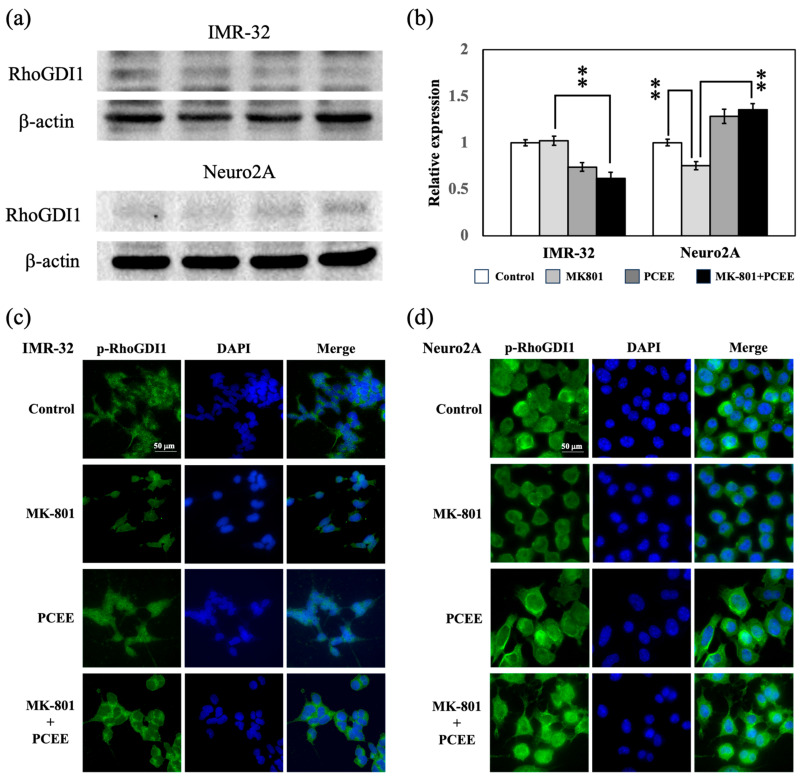
Effects of MK-801 and PCEE on the expression and phosphorylation of RhoGDI1 in IMR-32 and Neuro2A cells. Protein extracts collected from IMR-32 and Neuro2A cells treated with MK-801 and/or PCEE were examined for RhoGDI1 expression (**a**,**b**) through immunoblotting. RhoGDI1 phosphorylation was also examined via immunofluorescent staining in IMR-32 (**c**) and Neuro2A (**d**) cells. The bar charts were generated from triplicate Western blot data from three different batches of drug-treated cells. A *p* value less than 0.01 (**) from ANOVA followed by Dunnett’s test was considered significant. The image was visualized under a fluorescence microscope at 40× magnification.

RhoGDI1 is an upstream modulator of the RhoA/ROCK1 pathway [18]. The expressions of RhoGDI1 and p-RhoGDI1 were then examined in vitro in IMR-32 and Neuro2A cells by immunoblotting and immunofluorescence staining. Our results revealed that PCEE significantly reversed the effects of MK-801 on the expression of RhoGDI1 in IMR-32 and Neuro2A cells (*p* value < 0.01) (Figure 6a,b). The phosphorylation of RhoGDI1 can activate RhoGDI1 and cause it to dock on the inner side of the cell membrane. Immunofluorescence staining revealed that MK-801 reduced p-RhoGDI1 levels in IMR-32 and Neuro2A cells, and PCEE reversed the decrease in p-RhoGDI1 caused by MK-801 (Figure 6c,d).

### 3.4. MK-801 and PCEE Modulate the RhoA/ROCK1 Pathway and Downstream MLC2 and PFN1 Regulation

**Figure 7:**
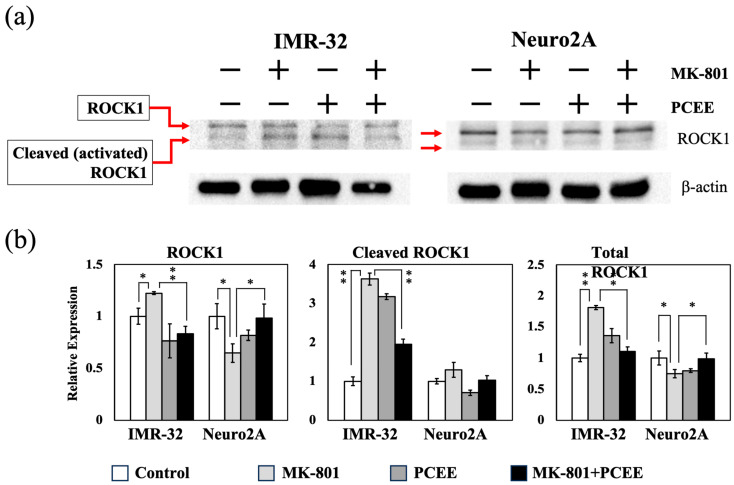
Effects of MK-801 and PCEE on the expression and activation of ROCK1 in IMR-32 and Neuro2A cells. (**a**) Protein extracts collected from IMR-32 and Neuro2A cells treated with MK-801 or/and PCEE were examined for the expression and cleavage of ROCK1 using immunoblotting. (**b**) Quantitative results of the expression of ROCK1, cleaved ROCK1, and total ROCK1. The bar charts were generated from triplicate Western blot data from three different batches of drug-treated cells. A *p* value less than 0.01 (**) or 0.05 (*) from ANOVA followed by Dunnett’s test was considered significant.

RhoA/ROCK1 signaling is an upstream regulator of MLC2. After activation, ROCK1 is cleaved to form activated ROCK1, which induces MLC2 phosphorylation [13]. The in vitro expression levels of ROCK1 and cleaved ROCK1 in IMR-32 and Neuro2A cells were measured by immunoblotting. MK-801 upregulated the expression of ROCK1, cleaved ROCK1, and total ROCK1 in IMR-32 cells. PCEE significantly reduced the MK-801-induced upregulation of ROCK1, cleaved ROCK1, and total ROCK1 (Figure 7a,b) (*p* value < 0.01).

Although the cleaved ROCK1 levels were comparable between the groups of Neuro2A cells, PCEE significantly downregulated the levels of ROCK1 and total ROCK1 in the MK-801 + PCEE group (*p* value < 0.05). These results suggested that PCEE inhibited the MK-801-induced activation of ROCK1.

**Figure 8:**
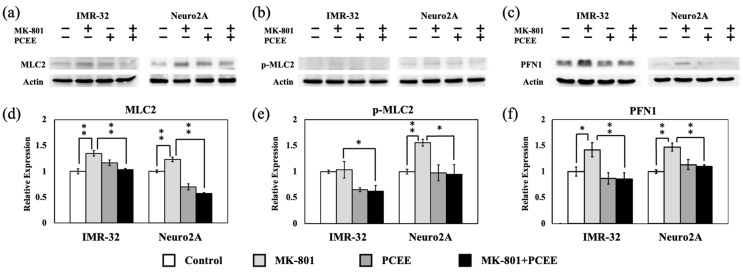
Effects of MK-801 and PCEE on the regulation of MLC2 and PFN1 in IMR-32 and Neuro2A cells. Protein extracts collected from IMR-32 and Neuro2A cells treated with MK-801 and/or PCEE were examined for the expression of MLC2 (**a**), phosphorylated MLC2 (p-MLC2) (**b**), and PFN1 (**c**), and the quantitative results are shown in (**d**–**f**). The bar charts were generated from triplicate Western blot data from three different batches of drug-treated cells. A *p* value less than 0.01 (**) or 0.05 (*) from ANOVA followed by Dunnett’s test was considered significant.

Phosphorylation of MLC2 modulates actin contractility and destabilization [14]. Therefore, we measured the levels of MLC2 and phosphorylated MLC2 (p-MLC2) by immunoblotting. Our results revealed that MK-801 stimulation upregulated MLC2 expression (Figure 8a,d). PCEE significantly mitigated the MK-801-induced increase in MLC2 expression in IMR-32 and Neuro2A cells (Figure 8a,d) (*p* value < 0.01). Furthermore, PCEE reduced the levels of p-MLC2 in the cells treated with MK-801 (Figure 8b,e), suggesting that PCEE regulated the MK-801-induced phosphorylation of MLC2. MK-801 increased PFN1 expression in IMR-32 and Neuro2A cells (Figure 8c,f), and this effect was significantly reversed by PCEE in IMR-32 and Neuro2A cells (*p* value < 0.01).

### 3.5. PCEE Reverses the Inhibitory Effect of MK-801 on Cell Mobility In Vitro

**Figure 9:**
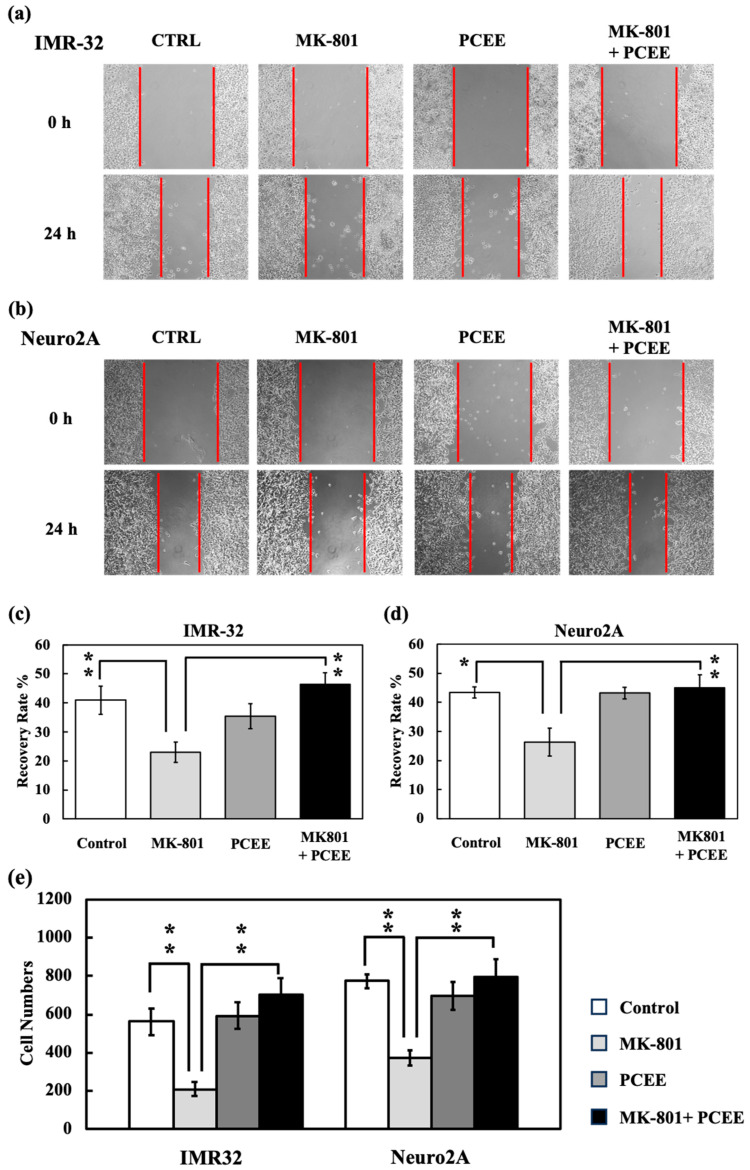
Recovery effect of PCEE on the reduction in cell mobility caused by MK-801 in IMR-32 and Neuro2A cells. Cell mobility was measured via wound healing and migration assays. In the wound healing assay, cell-free gaps in cultured IMR-32 (**a**) or Neuro2A (**b**) cells were observed and recorded at 0 h and 24 h. The recovery rate was calculated as the percentage reduction in the width of the cell-free gaps in IMR-32 (**c**) or Neuro2A (**d**) cells at 24 h compared with the cell-free gap at 0 h. In the migration assay (**e**), the migrated cells in each group were counted to indicate the cell migration ability. A *p* value of less than 0.01 (**) or 0.05 (*) was considered significant.

We also investigated the effects of PCEE on cell mobility via wound healing and migration assays. MK-801 reduced the level of wound healing in IMR-32 (Figure 9a,c) and Neuro2A (Figure 9b,d) cells. This inhibitory effect of MK-801 on wound healing was counteracted by PCEE. The migration assay data were compatible with the wound healing data. The level of cell migration in the MK-801 + PCEE group was significantly greater than that in the MK-801 group (Figure 9e). These results indicated that PCEE reversed the reduction in cell mobility caused by MK-801 stimulation.

### 3.6. PCEE Increases the Levels of F-Actin Condensation and Nucleation In Vitro

**Figure 10:**
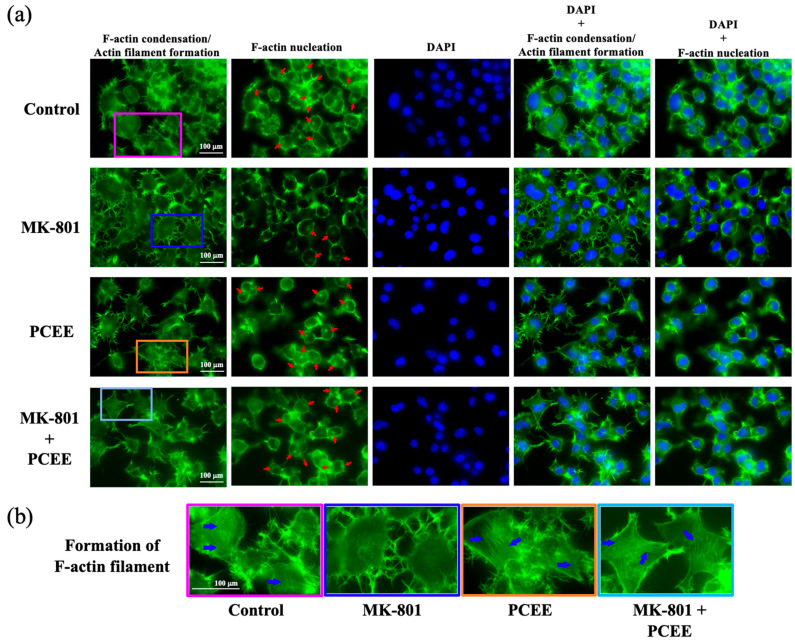
Effects of MK-801 and PCEE on F-actin filament condensation and nucleation in Neuro2A cells. F-actin filament condensation and nucleation were examined via immunofluorescence staining with phalloidin. (**a**) PCEE reversed the inhibitory effect of MK-801 on F-actin nucleation (red arrows) and (**b**) F-actin filament condensation (formation of actin filaments, blue arrows). The image was visualized under a fluorescence microscope at 40× magnification.

We measured the effects of PCEE on F-actin condensation and nucleation via phalloidin staining. F-actin condensation leads to actin filament formation, which is characterized by a thread-like pattern inside cells. Our results showed that MK-801 stimulation reduced the formation of F-actin filaments (Figure 10a,b). MK-801 also reduced actin nucleation (Figure 10a). Notably, the levels of F-actin filament formation and F-actin nucleation were greater in the MK-801 + PCEE group than in the MK-801 group. These results suggested that PCEE strongly counteracted the inhibitory effects of MK-801 on F-actin filament formation and F-actin nucleation. MK-801 and PCEE exerted similar regulatory effects on IMR-32 cells.

### 3.7. PCEE Mitigates MK-801-Induced Hyperactivity in Mice

**Figure 11:**
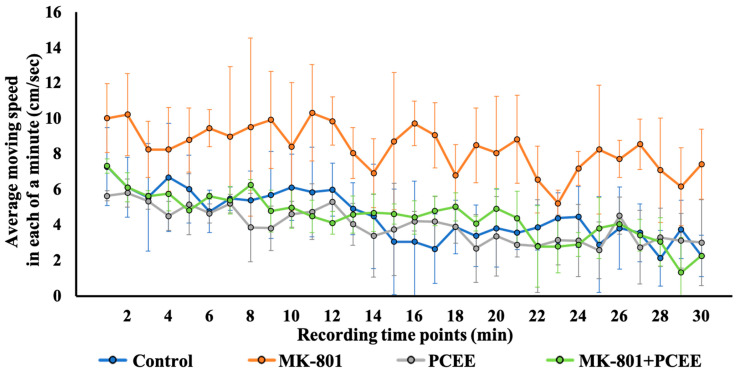
Restoration effect of PCEE on the MK-801-induced hyperactivity of C57BL/6 mice during the entire 30 min interval. In the open field test, the distance traveled by each mouse at each minute point within 30 min was recorded. The mice were randomly allocated into four groups, as illustrated in Figure 2. The average moving speed of the mice in each group (n = 3) was calculated and is shown as the mean ± SD.

Our in vitro data indicated that PCEE reversed the MK-801-induced modulation of the Rho signaling pathway. We sought to clarify the in vivo effects of PCEE in MK-801-treated mice. The behaviors of the C57BL/6 mice treated with MK-801 were assessed via an open field test. The movement speed of the mice in each group during the entire 30 min time interval was recorded. As shown in Figure 11, the moving speed of the mice in the MK-801 + PCEE group was significantly lower than that of the mice in the MK-801 group. MK-801 treatment obviously increased the movement speed of the mice at 0–30 and 10–20 min (Table 2), suggesting that locomotor hyperactivity was induced in the MK-801-stimulated mice. Therefore, PCEE mitigated the locomotor hyperactivity induced by MK-801. No significant difference in moving speed was found between the control, PCEE, and MK-801 + PCEE groups. These results demonstrated the significant recovery effect of PCEE (*p* value < 0.01) on MK-801-triggered locomotor hyperactivity in C57BL/6 mice.

### 3.8. PCEE Modulates the Expression of Rho Family Proteins in the PFC of C57BL/6 Mice

**Figure 12:**
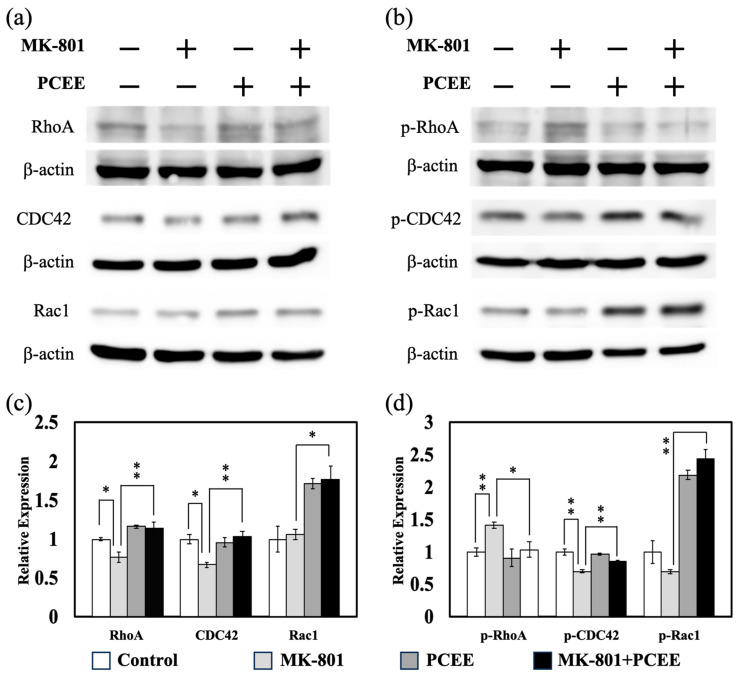
Effects of MK-801 and PCEE on Rho family protein expression and/or activation in the PFC of C57BL/6 mice. The expression levels of Rho family proteins (**a**) and phosphorylated Rho family proteins (**b**) were examined by immunoblotting, and the quantitative results are shown in (**c**,**d**). The mice were randomly allocated into four groups, as illustrated in Figure 2. A *p* value less than 0.01 (**) or 0.05 (*) from ANOVA followed by Dunnett’s test was considered significant.

After the open field test, the mice were sacrificed, and the PFC was collected to examine the in vivo expression of Rho signaling-related proteins. The expression of RhoA and CDC42, but not Rac1, was reduced in the PFC of the mice treated with MK-801 (Figure 12a,c). PCEE reversed the MK-801-induced reduction in RhoA and CDC42 expression in the PFC. Rac1 expression in the MK-801 + PCEE-treated group was significantly greater than that in the MK-801 group (Figure 12a,c). Furthermore, PCEE modulated the phosphorylation of Rho family proteins (Figure 12b,d).

**Figure 13:**
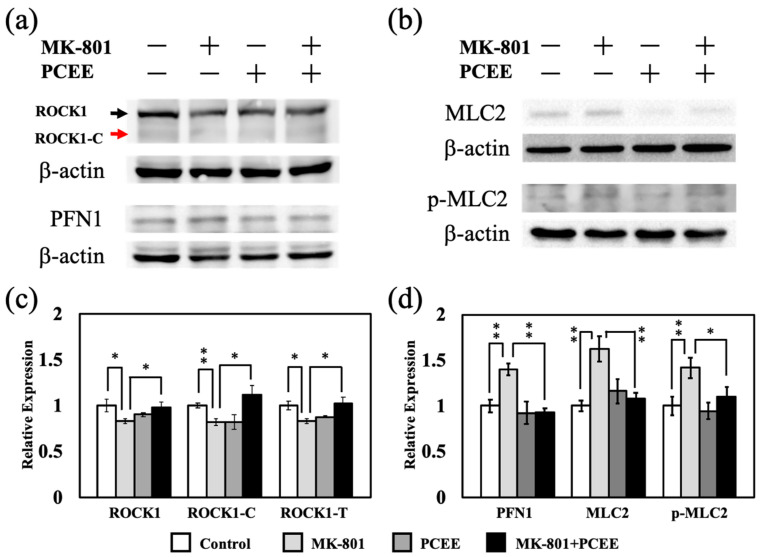
Effects of MK-801 and PCEE on ROCK1, MLC2, and PFN1 expression and/or activation in the PFC of C57BL/6 mice. The expression levels of ROCK1, cleaved ROCK1 (ROCK1-C), total ROCK1 (ROCK1-T), MLC2, p-MLC2 and PFN1 were examined (**a,b**), and the quantitative results are shown in (**c**,**d**). The mice were randomly allocated into four groups, as illustrated in Figure 2. A *p* value less than 0.01 (**) or 0.05 (*) from ANOVA followed by Dunnett’s test was considered significant.

It also reversed the MK-801-induced regulation of phosphorylated Rho family proteins expression in the PFC of the mice. Our data also revealed that MK-801 reduced ROCK1, cleaved ROCK1, and total ROCK1 levels, whereas PCEE reversed the MK-801-induced decrease in protein expression (Figure 13a,b). Furthermore, the expression of MLC2, p-MLC2, and PFN1 was increased by MK-801 treatment (Figure 13a,c). The effect of MK-801 on MLC2, p-MLC2, and PFN1 expression was counteracted by PCEE treatment.

### 3.9. Summary of MK-801/PCEE-Induced In Vivo and In Vitro Regulation of Rho Signaling-Related Proteins

We summarize the MK-801/PCEE-induced in vivo and in vitro regulation of Rho signaling-related proteins in Table 3. The same protein regulation trend in C57BL/6 mice or IMR-32 cells compared to Neuro2A cells are highlighted in bold.

## 4. Discussion

In this study, we demonstrated the effect of PCEE on MK-801-induced cytoskeletal reorganization, which may be linked to cellular functions, such as cell migration and neuronal plasticity. MK-801 reduced cell migration, F-actin condensation, and actin nucleation in vitro. Previous reports have shown that ketamine disrupts the polymerization of actin filaments, which partly supports our results [31]. Ketamine and MK-801 trigger the continuous excitation of neuronal cells in the brain, which may be accompanied by disturbances in neurotransmitter release, leading to psychosis-related presentations. Notably, PCEE reversed the effects of MK-801 on cytoskeletal dynamics, suggesting a role for PCEE in mitigating psychotic behavior. In the present study, PCEE reduced MK-801-induced locomotor hyperactivity in mice, as demonstrated by the reduced movement speed. Our results also demonstrated that the regulatory effects of MK-801 and PCEE on Rho family proteins were similar in vitro and in vivo. Thus, PCEE may regulate Rho signaling while easing MK-801-induced hyperactivity.

Our previous report revealed that antipsychotic drugs act by regulating Rho family proteins, suggesting that the Rho signaling pathway is a therapeutic target for psychosis [32]. In the present study, we identified the RhoA/ROCK1 signaling pathway as a possible mechanism underlying the effect of PCEE on the MK-801 model. In addition to MLC2, PFN1 is another downstream effector of RhoA/ROCK1 signaling [33]. PFN1 activity has been linked to the migration ability of neuronal cells and glial cells in various studies [15,34]. In the present study, increased PFN1 expression was accompanied by the inhibitory effect of MK-801 on RhoA/ROCK1 expression, suggesting that there might be a feedback mechanism involving PFN1. A previously published report supports the notion that a feedback mechanism exists between PFN, actin, and ROCK signaling [35]. Other Rho family proteins, such as Rac1 and CDC42, are also modulated by PCEE. Taken together, the results of these previous studies and the present study suggested that PCEE mitigated MK-801-induced psychotic behavior by modulating the Rho signaling pathway. A previous study reported that PCEE regulates the cytoplasmic free calcium concentration in rat brain neurons [36]. Similarly, a ketamine-triggered imbalance in Ca^2+^ homeostasis has been linked to subsequent neuronal function [37]. Therefore, considering that calcium is a key regulator of RhoGDI1 activation, we hypothesize that the effect of PCEE on the Rho signaling pathway is mediated by calcium. However, the exact mechanism by which PCEE activates RhoGDI1 requires further investigation.

Our in vitro data revealed some inconsistent results between IMR-32 and Neuro2A cells. In IMR-32 cells, PCEE mitigated RhoA phosphorylation, ROCK1 cleavage, and MLC2 phosphorylation. However, in Neuro2A cells, PCEE increased RhoA phosphorylation and ROCK1 cleavage and reduced MLC2 phosphorylation. We speculate that the differences between IMR-32 and Neuro2A cells may have contributed to this inconsistency. Another possible explanation for this inconsistency is feedback inhibition in the phosphorylation cascade. Although our data are clear, some limitations remain in this study. Firstly, only a single dose of PCEE was administered. However, whether PCEE exerts a dose-dependent effect on locomotor hyperactivity in MK-801-stimulated mice remains unknown. Secondly, the roles of other possible upstream regulators of the RhoA/ROCK1 pathway, such as cytokines, in the action of PCEE remain unclear.

Currently, antipsychotic drugs are the mainstream therapies for psychiatric diseases. However, the molecular mechanisms underlying the action of antipsychotic drugs are not fully understood. Although antipsychotic drugs are effective for most patients with psychosis, they have various side effects [38]. Our findings provide evidence supporting a new potential therapy targeting Rho GTPases in psychotic diseases. In addition, we did not observe a significant difference between the control and PCEE groups in most experiments. In Neuro2A cells, the levels of migration, F-actin condensation and nucleation, and the levels of Rho protein, phosphorylated Rho protein, and p-MLC2 in the PCEE group were comparable to those in the control group (Table 2). These results suggest that PCEE restored MK-801-induced changes to similar levels as the control group in most tests, which could be the most critical effect of PCEE on restoring the normal function of cells. We can expect that the less PCEE effects Rho signaling regulation, the fewer unpredictable effects will be caused on cells. Interestingly, we found similar regulations of protein downstream in the Rho signaling (ROCK1, MLC-2, p-MLC2 and PFN1) between C57BL/6 mice and Neuro2A cells of the same species. We also revealed similar regulations of protein upstream in the Rho signaling (RhoGDI1 phosphorylation and Rho protein expression) between IMR-32 mice and Neuro2A cells of the same cell type. These observations suggest that the effects of MK-801 and PCEE on cells might vary according to cell types and animal species.

In addition, the locomotor activity of C57BL/6 mice was also not altered by treatment with PCEE alone. These findings suggest that PCEE provides recovery effects but minimal regulatory effects on Rho signaling in neuronal cells and the PFC of C57BL/6 mice. These observations suggest that PCEE has few side effects when used as a therapeutic agent. The differences in Rho family and Rho signaling protein regulation between C57BL/6 mice and Neuro2A cells may be caused by a combination of protein modulation of various cell types in the PFC of the mice. Furthermore, PCEE has anti-inflammatory and immunomodulatory properties [39]. Psychotic diseases are usually accompanied by chronic inflammation [40]. From the perspective of immunomodulation, PCEE shows promise as a potential herbal medicine and natural alternative for the adjunctive or complementary treatment of psychosis.

The doses of PCEE used for treating mice (10 mg/Kg/day) correspond to 1.68 mg/Kg for TA, 2.93 mg/Kg for PA, 1.18 mg/Kg for DTA, and 0.87 mg/Kg for PPA a day. As mentioned in Section 2.1, *P. cocos* concentrated herbal extract was manufactured as a dried powder. The suggested therapeutic dose of *P. cocos* concentrated herbal extract taken for daily use is 1.2–3 g which corresponds to 20.2–50.4 mg for TA, 35.2–88 mg for PA, 14.1–35.3 mg for DTA, and 10.4–26.1 mg for PPA. The equivalent for a human adult with a body weight of 65 Kg is 0.31–0.776 mg/Kg for TA, 0.542–1.354 mg/Kg for PA, 0.217–0.543 mg/Kg for DTA, and 0.16–0.401 mg/Kg for PPA. The doses of TA, PA, DTA, and PPA used to treat mice are about 2.17- to 5.14-times higher than those used for treating a 65 Kg human. It is worth mentioning that this corresponding fold in humans will vary depending on their body weight and also on the uptake efficiency of active compounds from PCEE in their body.

PA was investigated for its potential to inhibit migration/invasion and reduce RhoA expression in gallbladder cancer cells [41]. PA and PPA were also demonstrated to reduce cell migration and invasion in various cancer cells [42,43]. Interestingly, the Rho signaling regulation, migration ability, and cytoskeleton remodeling of IMR-32/Neuro2A cells were not affected by PCEE treatment in this study. These observations might be caused by the chronic effects (28 days) of PCEE on cells, which suggested that the acute effects of PCEE and its ingredients were different from the chronic effects on cells. At present, there are still few research results mentioning the role of PCEE and even its ingredients (such as PA, TA, DTA, and PPA) in Rho signaling and cytoskeleton reorganization. It will be necessary to conduct more studies to understand the roles of *P. cocos* ingredients in regulating cell function by modulating Rho signaling.

Structural and functional modifications in neuronal circuits can be caused by chronic administration of antipsychotics, including changes in dendritic spine morphology and synaptic integrity [44,45]. This is potentially due to non-specific modulation of intracellular signaling cascades, such as the Rho/ROCK pathway [46]. Our findings demonstrated that PCEE is capable of normalizing aberrant Rho signaling activity caused by MK-801 without interfering with the function of the physiological Rho pathway under basal conditions. Rho GTPases, such as RhoA, Rac1, and CDC 42, play an important role in regulating cytoskeletal dynamics, spine architecture, and synaptic flexibility [47,48]. Dysregulation of these signaling molecules has been implicated in the pathogenesis of schizophrenia and other neurodevelopmental disorders. However, direct pharmacological targeting of Rho signaling has been limited due to the potential for off-target effects and toxicity [49]. PCEE has the potential to be a promising therapeutic agent with an improved safety profile due to its ability to selectively restore Rho pathway homeostasis in pathological conditions.

## 5. Conclusions

This study demonstrates that PCEE regulates RhoA/ROCK1 signaling to modulate cytoskeletal dynamics in the Neuro2A cell and to mitigate MK-801-induced locomotor hyperactivity in mice. Our results revealed the role of the RhoA/ROCK1 pathway in psychosis and the potential therapeutic effect of PCEE on psychotic behavioral changes. Also, PCEE represents a safer and more targeted alternative to conventional antipsychotic drugs by selectively modulating pathological signaling without affecting normal Rho pathway activity. Future research should explore the molecular mechanisms underlying the differential effects of ingredients in *P. cocos* on regulating Rho signaling and their potential therapeutic applications in neuropsychiatric disease.

## Figures and Tables

**Table 1:** Quantification of active compounds in PCEE using HPLC.

Active Compound	Stand Curve 1, 5, 10, 50, and 100 mg/mL	R^2^	mg/100 mg PCEE			Mean ± S.D. (mg/100 mg PCEE)
			Batch1	Batch2	Batch3	
TA	Y = 8494.41X + 950.361	0.99996	16.188	17.411	16.809	16.803 ± 0.612
PA	Y = 6765.41X − 415.930	0.99961	28.266	30.427	29.335	29.343 ± 1.081
DTA	Y = 21,148.8X + 3179.20	0.99996	11.385	12.196	11.736	11.772 ± 0.407
PPA	Y = 16,091.0X + 856.040	0.99993	8.387	9.008	8.68	8.692 ± 0.311

**Table 2:** PCEE reduces the MK-801-induced hyperactivity of C57BL/6 mice.

Time Interval of Test	Group (*n* = 3)	Mean of Moving Speed (cm/s)	Standard Deviation (SD) of Moving Speed	*p* Value
0–30 min	Control	4.5078	2.1682	<0.01 ^#^
	MK-801	8.3722	2.3778	
	PCEE	3.9898	1.5851	<0.01 ^‡^
	MK-801 + PCEE	4.4522	1.5314	
10–20 min	Control	4.1233	2.2342	<0.01 ^#^
	MK-801	8.6100	2.2233	
	PCEE	3.9680	1.565	<0.01 ^‡^
	MK-801 + PCEE	4.5867	0.782	

^#^ MK-801 treated mice compared with control mice.; ^‡^ MK-801 treated mice compared with MK-801 + PCEE treated mice.

**Table 3:** Summary of MK-801/PCEE-induced in vivo and in vitro regulation of Rho signaling-related proteins.

	C57BL/6			NuoroA			IMR-32		
	MK-801	PCEE	MK801 + PCEE	MK-801	PCEE	MK801 + PCEE	MK-801	PCEE	MK801 + PCEE
**RhoGDI1**	**D**	N	N	D	I	I	N	D	D
**p-RhoGDI1**	-	-	-	D	N	N	**D**	**N**	**N**
**RhoA**	**D**	I	**I**	D	N	I	**D**	**N**	**I**
**CDC42**	**D**	**N**	N	D	N	I	**D**	**N**	N
**Rac1**	N	I	I	D	N	N	**D**	**N**	**N**
**p-RhoA**	I	**N**	N	N	N	I	I	D	N
**p-CDC42**	D	**N**	**N**	I	N	N	D	D	D
**p-Rac1**	**D**	I	I	D	N	N	I	D	**N**
**ROCK1**	**D**	**D**	**N**	D	D	N	I	I	**N**
**MLC2**	**I**	**D**	N	I	D	D	**I**	N	N
**p-MLC2**	**I**	**N**	**N**	I	N	N	**N**	D	D
**PFN1**	**I**	**N**	**N**	I	N	N	**I**	**N**	**N**

D: decreased expression compared with the control; I: increased expression compared with the control; N: comparable expression compared with the control; -: not tested. The bold style highlights the same protein regulation trend in C57BL/6 mice or in IMR-32 cells compared with Neuro2A cells.

## Data Availability

The data that support the findings of this study are available from the corresponding author upon reasonable request.

## Abbreviations

The following abbreviations are used in this manuscript:

PCEE	Poria cocos ethanol extract
NMDA	N-methyl-D-aspartate
RhoGDI1	Rho GDP dissociation inhibitor 1
RhoA	Rho family protein (Ras homolog family member A
CDC42	cell division control protein 42 homolog
Rac1	Ras-related C3 botulinum toxin substrate 1
ROCK1	Rho-associated, coiled-coil-containing protein kinase 1
MLC2	myosin light chain 2
PFN1	profilin-1
PA	pachymic acid
TA	tumulosic acid
DTA	dehydrotumulosic acid
PPA	polyporenic acid C
FBS	fetal bovine serum
PFC	prefrontal cortex
